# Supplementary material for: Colorectal Cancer Risk in Patients with Hemorrhoids: A 10-Year Population-Based Retrospective Cohort Study
Source: Int J Environ Res Public Health. 2021 Aug 16;18(16):8655. doi: 10.3390/ijerph18168655 (PMC8394877; doi:10.3390/ijerph18168655)
Supplement: Supplementary file 1 [file ijerph-18-08655-s001.zip › ijerph-1244969-supplementary.pdf]

International Journal of
Environmental Research and Public Health

MDPI

Table S1. Demographic characteristics and comorbidities in patients with and without hemorrhoids who were matched by sex and age.

Variable	Hemorrhoids No n = 150,852	Hemorrhoids Yes n = 37,713	Standardized Difference §
Sex	n (%)	n (%)	
Female	69,104 (45.8)	17,276 (45.8)	0.000
Male	81,748 (54.2)	20,437 (54.2)	0.000
Age, mean (SD)	46.8 (16.2)	47.3 (15.9)	0.023
≤49 years	91,328 (60.5)	22,832 (60.5)	0.000
50–64	34,968 (23.2)	8742 (23.2)	0.000
65+	24,556 (16.3)	6139 (16.3)	0.000
Comorbidity			
IBD	1173 (0.78)	607 (1.61)	0.08
Hypertension	33,136 (22.0)	10,093 (26.8)	0.11
Diabetes	9569 (6.34)	2354 (6.24)	0.004
Hyperlipidemia	20,126 (13.3)	7512 (19.9)	0.18
Stroke	3701 (2.45)	941 (2.50)	0.003
Congestive heart failure	3546 (2.35)	1173 (3.11)	0.047
Cancer, except colorectal cancer	1959 (1.30)	845 (2.24)	0.07
Colorectal adenomas	221 (0.15)	559 (1.48)	0.15
Obesity	530 (0.35)	194 (0.51)	0.03
Appendectomy	1449 (0.96)	489 (1.30)	0.03
PLA	97 (0.06)	24 (0.06)	0.000

§ A standardized mean difference of ≤0.10 indicates a negligible difference between the two cohorts. IBD, inflammatory bowel disease; PLA, pyogenic liver abscess.

Table S2. Demographic characteristics and comorbidities in patients with and without hemorrhoids who were matched by propensity score with colonoscopy included.

Variable	Hemorrhoids No n = 34,712	Hemorrhoids Yes n = 34,712	Standardized Difference §
Sex	n (%)	n (%)	
Female	16,217 (46.7)	15,932 (45.9)	0.000
Male	18,495 (53.3)	18,780 (54.1)	0.000
Age, mean (SD)	47.6 (15.9)	47.1 (15.9)	0.023
≤49 years	20,274 (58.4)	21,208 (61.1)	0.000
50–64	8592 (24.8)	7875 (22.7)	0.000
65+	5846 (16.8)	5629 (16.2)	0.000
Comorbidity			
IBD	495 (1.43)	485 (1.40)	0.077
Hypertension	9217 (26.6)	8937 (25.8)	0.112
Diabetes	2363 (6.81)	2225 (6.41)	0.004
Hyperlipidemia	6664 (19.2)	6308 (18.2)	0.177
Stroke	901 (2.60)	882 (2.54)	0.003
Congestive heart failure	1066 (3.07)	1032 (2.97)	0.047
Cancer, except colon cancer	746 (2.15)	696 (2.01)	0.071
Colorectal adenomas	672 (1.94)	784 (2.26)	0.149
Obesity	187 (0.54)	161 (0.46)	0.025

Appendectomy	774 (2.23)	740 (2.13)	0.032
PLA	21 (0.06)	24 (0.07)	0.000
HBV	1359 (3.92)	1324 (3.81)	0.005
HCV	471 (1.36)	464 (1.34)	0.002
COPD	3531 (10.2)	3388 (9.76)	0.014
Alcohol-related illness	1254 (3.61)	1218 (3.51)	0.006
Chronic pancreatitis	60 (0.17)	52 (0.15)	0.006
Colonoscopy	9272 (26.7)	9268 (26.7)	0.000

§ A standardized mean difference of ≤0.10 indicates a negligible difference between the two cohorts. SD, standard deviation; IBD, inflammatory bowel disease; PLA, pyogenic liver abscess; HBV, hepatitis B virus; HCV, hepatitis C virus; COPD, chronic obstructive pulmonary disease.

Table S3. Overall incidence rates and hazard ratios of colorectal cancer of the hemorrhoids and comparison cohorts in first, second, and third sets of cohorts.

Study Set	Hemorrhoids						Crude HR (95% CI)	Adjusted HR (95% CI)
	No			Yes				
	Event	PY	Rate #	Event	PY	Rate #		
First	563	1,033,921	0.54	341	263,533	1.29	2.37 (2.07, 2.71) *	2.28 (1.99, 2.62) *
Second	138	25,572	0.54	337	261,466	1.29	2.39 (1.96, 2.93) *	2.18 (1.78, 2.67) *
Third	384	372,122	1.62	290	237,435	1.22	0.75 (0.65, 0.88) *	0.88 (0.69, 0.94) *

Rate #: incidence rate per 1000 person-years; PY, person-years; HR, hazard ratio. Adjusted HR : estimated after controlling for sex, age, and comorbidity. Comorbidity: patients with any one of the comorbidities (IBD, hypertension, diabetes, hyperlipidemia, stroke, congestive heart failure, obesity, PLA, HBV, HCV, COPD, alcohol-related illness, and chronic pancreatitis) were assigned to the comorbidity group. * $p < 0.001$.

Table S4. Number of colonoscopies received before and after baseline compared between cohorts with and without hemorrhoids.

Variable	Hemorrhoids				*p*-Value
	No *n* = 36864		Yes *n* = 36864		
**Colonoscopy**	*n*	Rate	*n*	Rate	
**Before**					<0.001
0	35,993	97.6	32,110	87.1	
1	732	1.99	3926	10.7	
2	102	0.28	614	1.67	
≧3	37	0.10	214	0.58	
**After**					<0.001
0	35,115	95.3	26,338	71.5	
1	1391	3.77	6994	19.0	
2	253	0.69	2193	5.95	
≧3	105	0.28	1339	3.63	

Table S5. Incidences and hazard ratios of colorectal cancer associated with hemorrhoid surgery.

Variables	*n*	Event	Rate #	Crude HR (95% CI)	Adjusted HR (95% CI)
Non-hemorrhoids	36,864	131	0.53	1.00	1.00
Hemorrhoids					
Surgery					
**No**	27,783	280	1.46	2.76 (2.24, 3.39) **	2.31 (1.88, 2.85) **
**Yes**	9081	50	0.74	1.38 (0.99, 1.91)	1.65 (1.19, 2.29) *

Rate #: incidence per 1000 person-years; HR, hazard ratio. Adjusted HR : controlling for sex and age. *$p < 0.01$, **$p < 0.001$.

Table S6. Incidence and hazard ratios of colorectal cancer associated with polyp or hemorrhage compared between the cohorts with and without hemorrhoids.

Variable	Hemorrhoids						Crude HR (95% CI)	Adjusted HR (95% CI)
	No			Yes				
	Event	PY	Rate #	Event	PY	Rate #		
Polyp or hemorrhage of rectum/anus								
**No**	124	245,766	0.50	266	228,979	1.16	2.29 (1.85, 2.84) *	2.09 (1.69, 2.59) *
**Yes**	7	1657	4.22	64	29,914	2.14	0.50 (0.23, 1.10)	0.59 (0.27, 1.31)
Hemorrhage of gastrointestinal tract, unspecified								
**No**	102	234,739	0.43	226	219,621	1.03	2.35 (1.86, 2.97) *	2.03 (1.60, 2.59) *
**Yes**	29	12,684	2.29	104	39,273	2.65	1.16 (0.77, 1.74)	1.27 (0.83, 1.94)

Rate #: incidence rate per 1000 person-years; PY person-years, HR hazard ratio. Adjusted HR : adjusted for age, sex and comorbidity. *$p < 0.001$.
